# Sensitivity to temporal parameters of intraneural tactile sensory feedback

**DOI:** 10.1186/s12984-020-00737-8

**Published:** 2020-08-15

**Authors:** Giacomo Valle, Ivo Strauss, Edoardo D’Anna, Giuseppe Granata, Riccardo Di Iorio, Thomas Stieglitz, Paolo Maria Rossini, Stanisa Raspopovic, Francesco Maria Petrini, Silvestro Micera

**Affiliations:** 1 The BioRobotics Institute and Department of Excellence in Robotics and AI, Scuola Superiore Sant’Anna, Pisa, Italy; 2 Laboratory for Neuroengineering, Department of Health Sciences and Technology, Institute for Robotics and Intelligent Systems, ETH Zürich (ETH), 8092 Zürich, Switzerland; 3 Bertarelli Foundation Chair in Translational Neuroengineering, Centre for Neuroprosthetics and Institute of Bioengineering, School of Engineering, École Polytechnique Fédérale de Lausanne (EPFL), Lausanne, Switzerland; 4 Department of Physical Medicine and Rehabilitation, University of Pittsburgh, Pittsburgh, PA 15213 USA; 5 Institute of Neurology, Catholic University of The Sacred Heart, Policlinic A. Gemelli Foundation, Roma, Italy; 6 Laboratory for Biomedical Microtechnology, Department of Microsystems Engineering–IMTEK, Bernstein Center, BrainLinks-BrainTools Cluster of Excellence, University of Freiburg, D-79110 Freiburg, Germany; 7 Area of Neuroscience, IRCCS San Raffaele Pisana, Rome, Italy; 8 SensArs Neuroprosthetics, CH-1004 Lausanne, Switzerland

**Keywords:** Neural sensory feedback, Intraneural interface, Neural stimulation, Upper limb amputees

## Abstract

**Background:**

Recent studies have shown that neural stimulation can be used to provide artificial sensory feedback to amputees eliciting sensations referred on the amputated hand. The temporal properties of the neural stimulation modulate aspects of evoked sensations that can be exploited in a bidirectional hand prosthesis.

**Methods:**

We previously collected evidence that the derivative of the amplitude of the stimulation (intra-digit temporal dynamics) allows subjects to recognize object compliance and that the time delay among stimuli injected through electrodes implanted in different nerves (inter-digit temporal distance) allows to recognize object shapes. Nevertheless, a detailed characterization of the subjects’ sensitivity to variations of intra-digit temporal dynamic and inter-digit temporal distance of the intraneural tactile feedback has not been executed. An exhaustive understanding of the overall potentials and limits of intraneural stimulation to deliver sensory feedback is of paramount importance to bring this approach closer and closer to the natural situation. To this aim, here we asked two trans-radial amputees to identify stimuli with different temporal characteristics delivered to the same active site (intra-digit temporal Dynamic Recognition (DR)) or between two active sites (inter-digit Temporal distance Recognition (TR)). Finally, we compared the results achieved for (simulated) TR with conceptually similar experiments with real objects with one subject.

**Results:**

We found that the subjects were able to identify stimuli with temporal differences (perceptual thresholds) larger than 0.25 s for DR and larger than 0.125 s for TR, respectively. Moreover, we also found no statistically significant differences when the subjects were asked to identify three objects during simulated ‘open-loop’ TR experiments or real ‘closed-loop’ tests while controlling robotic hand.

**Conclusions:**

This study is a new step towards a more detailed analysis of the overall potentials and limits of intraneural sensory feedback. A full characterization is necessary to develop more advanced prostheses capable of restoring all lost functions and of being perceived more as a natural limb by users.

## Background

In the last decades, several research groups have shown the potential of neural stimulation to restore somatotopic sensations referred to the phantom hand after upper limb amputation by means of electrodes [1] being directly connected to the afferent fibers of the peripheral nerves [2–11]. Restoring sensory feedback using direct nerve stimulation improved prosthesis control [6, 9, 11], embodiment [11–14], time use [15], reduced phantom limb abnormal representations [11, 12, 15] and phantom limb pain [6, 13, 16] even in long-term implantations [6, 9, 15, 17].

Interestingly, the temporal parameters of neural stimulation are crucial to optimally exploit this kind of technology. Indeed, the modulation of neural stimulation at temporal, spatial, and intensity levels allowed to control grasping force [6, 7, 10], to perceive more natural sensations [11], to discriminate textures [18, 19], and to identify the physical properties of objects such as compliance and shape [7, 10, 20]. In particular, in order to recognize three objects with different compliances and shapes, the subject exploited the intra-digit and inter-digit temporal differences of the neural stimulation, respectively [7]. When the prosthetic hand was closed, the digits entered in contact with the grasped object at different timings allowing the subject to identify the object shape (inter-digit temporal distance) by using the time delay between different channels of stimulation (multichannel stimulation). On the other hand, when the prosthesis was closed by the subject on a soft object, the pressure applied by the digits increased more slowly compared to a hard object, and proportionally the intensity of the sensation stimulation-driven (intra-digit temporal dynamics).

However, a detailed characterization of the perceptual sensitivity of the subjects to variations of such intraneural stimulation temporal parameters has not been yet performed. This analysis is important to understand the potentials and limits of this approach and to develop more effective sensory feedback approaches. Reaching the spatial and temporal sensitivity of the human hand through an optimally designed neural stimulation will make possible to fully replace all the missing sensory information to amputees.

**Fig. 1:**
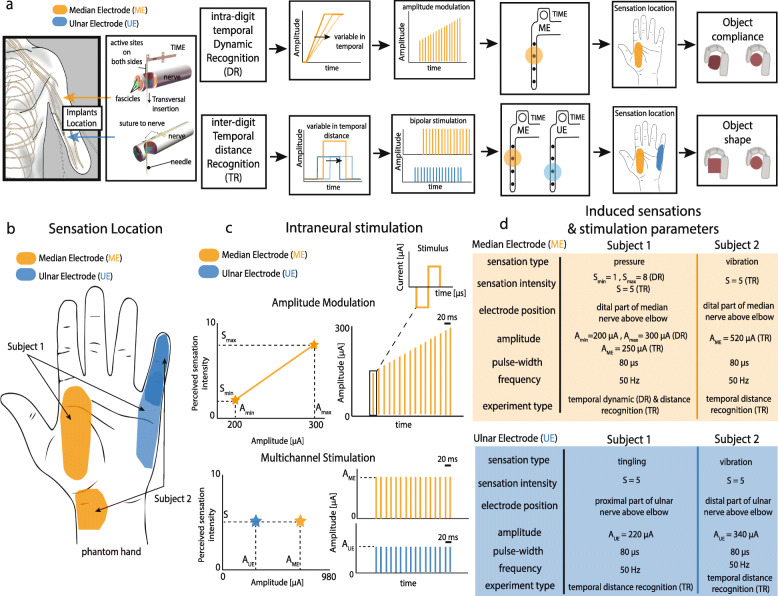
Intraneural sensory feedback to encode objects compliance and shape. **a** Subjects were implanted with four intraneural electrodes (TIME) in median and ulnar nerves (ME and UE) and were involved in two tasks: intra-digit temporal Dynamic Recognition (DR) and inter-digit Temporal distance Recognition (TR) tasks. In these tasks, the subjects have to recognize stimulation pattern different in temporal-distance and temporal-dynamic. These cues are exploited by subjects in closed-loop to encode objects shape and compliance. **b** Two active sites eliciting two locally-separated sensations on the phantom hand were considered for both subjects. **c** Schematic representation of the intraneural stimulation adopted. In DR, the amplitude of the intra-digit neural stimulation was modulated between Smin and Smax being related to the sensation intensity. In TR, the inter-digit temporal distance between two active sites stimulation (multichannel stimulation) was changed. **d** Induced sensation and stimulation parameters were reported for ME and UE for Subject 1 and 2

To this aim, in the present paper, we investigated the sensitivity to temporal parameters (intra-digit temporal dynamic and inter-digit temporal distance) of intraneural tactile sensory feedback. We implanted two upper limb amputees with four TIMEs (Transversal Intrafascicular Multichannel Electrodes) [21] in their upper arm nerves, and we characterized the sensitivity of the subjects to different simulated compliances (intra-digit temporal dynamic recognition, DR) and shapes (inter-digit temporal distance recognition, TR) elicited through intraneural stimulation (Fig. 1a).

Then, we performed a characterization of the temporal parameters to better understand the sensory processing of artificial sensory signals; on the other hand, we evaluated the ability of the participants to integrate and exploit this sensory feedback to distinguish relevant object characteristics. Finally, we provided a preliminary comparison between simulated and real-life TR experiments.

## Methods

### Subject recruitment

Two left-handed trans-radial amputees were involved in the clinical investigation. Subject 1 was a 48-year-old female with a traumatic trans-radial amputation of the distal left forearm (her dominant hand), which occurred 23 years before her enrollment in the trial. She was implanted in June 2016 [6]. Subject 2 was a right-handed, 53-year-old female trans-radial (distal two-thirds of the left forearm) amputee. The amputation occurred in December 2015, following a traumatic accident at work. She was implanted in June 2017 [6]. The subjects were enrolled for a period of approximately 6 months, during which experimental sessions were randomized. The data reported in the current manuscript were obtained on 2nd and 3rd month after implant for Subject 1 and between 3rd and 4th months after implant for Subject 2.

For personal reasons, Subject 2 decided not to participate in the DR experiments taking part just in a limited number of other procedures.

### Surgical procedure

During general anesthesia, through a 15-cm-long skin incision on the left arm, the median and ulnar nerves were exposed to implant a proximal and a distal TIME [21] in each nerve. The electrodes were implanted transversally inside the nerve, in order to be near to sensory fibers [22]**.** The surgical approach to implant TIMEs is reported in detail elsewhere [7]. Each TIME had 14 active sites usable to deliver the electrical stimulation. Stimulation pulses were delivered via percutaneous wires. After 180 days, under an operating microscope (Pentero, Carl ZEISS AG, DE), the four microelectrodes were removed, in accordance with the protocol and the obtained permissions.

### Sensation characterization

After the implantation, each channel of all the TIMEs was connected to a commercial neural stimulator (Ripple LLC, USA – see below).

A sensation characterization (or mapping) procedure was performed to explore the subjects’ sensation related to the stimulation from different electrodes and active sites (same procedure as in [6, 10]). The subjects were asked to report the location, extent, type, and strength of the generated sensations whenever they perceived them. The sensation strength (S) was reported on a scale between 0 (no-sensation) and 10 (maximal sensation intensity still acceptable but below the pain level).

While the stimulation amplitude was increased, the perceived sensation maintained the same properties (e.g. type), but its perceived intensity was increased as well, following a relationship already described in [3, 10].

The stimulation was triggered by using a custom-made Matlab (R2016b, The Mathworks, US) program in an ‘open-loop’ setup (i.e., the subject received the sensory feedback with no control of the hand prosthesis). This GUI also acquired the reports from the subjects, as described in [6].

The stimulator delivered in turn through each active site separately, 2-s trains of electrical current. Charge-balanced, biphasic, cathodic-first, rectangular stimulation pulses were applied versus a ground electrode integrated in the TIME. Each train was delivered with the same parameters (pulse width, amplitude, inter-pulse interval), then the amplitude was increased by the minimum step, after a pause of 2 s, to give the subject the time to understand and to answer (i.e., in the case of threshold perception). In particular, we first set the pulse width at minimal value (e.g., 20 μs, chosen in value between 10 μs and 120 μA, with a resolution of 10 μs) and increased the stimulation amplitude (from 10 μA to 980 uA, with a resolution of 20 μA) in order to find the perceptual thresholds [10, 23]. Then, if the perceptual threshold was not found, we repeated the procedure with a higher pulse width (eg. 30 μs). The perceptual threshold was the average of the charges at which the minimum sensation was reported by the subject.

At the end of the sensation characterization procedure, we found all the charges necessary to reach the perceptual thresholds for each active site. The amplitude ramp continued until reaching the maximal sensation intensity reported by the subjects (as the level below the pain threshold).

Additionally, we performed a multichannel stimulation (that would have been used for TR tests – see below) to characterize the sensation thresholds on two electrode channels simultaneously (as reported in [23]). In this protocol, trains of stimulation with increasing amplitude were delivered simultaneously from two active sites of interest. The threshold of the channels in the multichannel stimulation was the value of charge at which all the channels themselves elicited a sensation of minimum intensity at the same time. To determine such values, the minimum amplitude of stimulation of all the channels was manually adjusted. As in single channel configuration the single values of stimulation were delivered at least 3 times.

The results of the single-channel and multi-channel characterization procedures are described in [6, 23]. For this study, two active sites of two electrodes (one in the median nerve and one in the ulnar nerve) were chosen to elicit two well-perceivable, stable, and spatially-separated sensations referred on the phantom hand (Fig. 1).

The pulse amplitude of the adopted active sites varied between 200 μA and 520 μA depending by task and subject *(*Fig. 1d*),* while the pulse width was fixed to 80 μs, as was the train frequency (50 Hz, as in [7]). The injected charge remained always below the safety limit [21].

### Neural stimulator

Ripple Grapevine LCC (Neural Interface Processor - NIP, Ripple Grapevine US) was used to induce electrical stimulation of the TIME active sites. The neural stimulator was used to search for thresholds, check impedances, support real-time control of the stimulation (amplitude, pulse-width and frequency) using embedded safety procedures (limitation of 120 nC injected as preset maximum safe charge injection limit of every active site on the TIME) and provide a biphasic, balanced charged waveform.

### Intra-digit temporal dynamic recognition (DR)

**Fig. 2:**
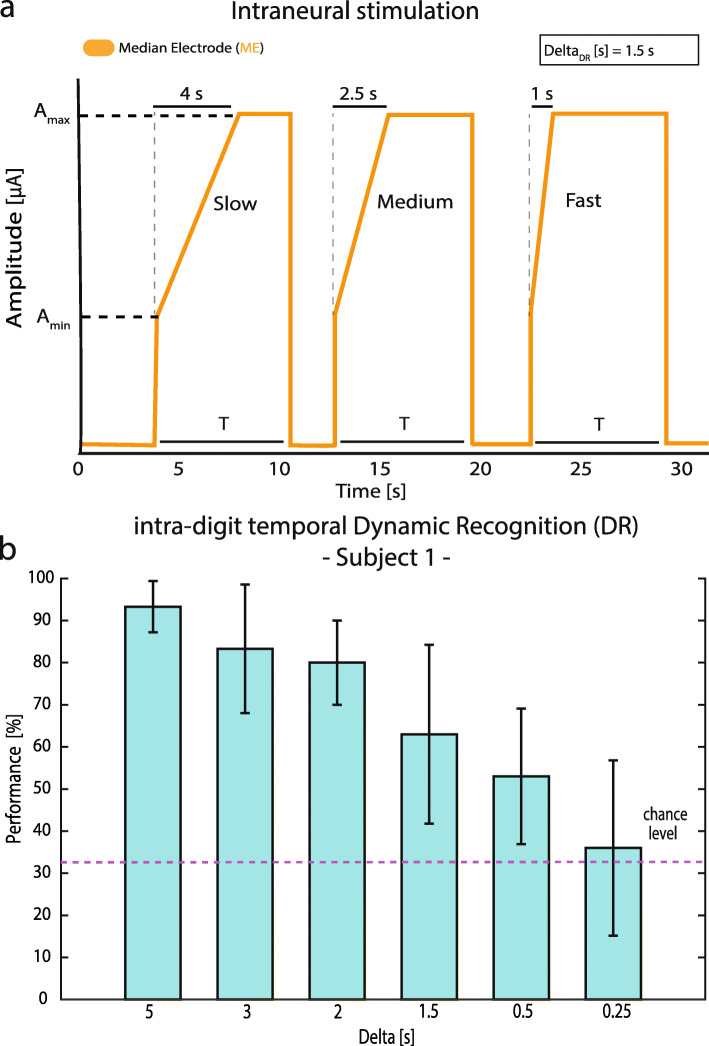
Intra-digit temporal Dynamic Recognition (DR) task to determine the sensitivity to intra-digit temporal dynamic of intraneural stimulation. **a** In DR the amplitude of intraneural stimulation was modulated between A_min_ and A_max_ following different temporal dynamics separated by Delta_DR_. **b** Performances according to different Delta are reported for Subject 1 (*N* = 6 sessions each of 90 repetitions). Means ± STD are reported. Delta_DR_ = 0.25 s resulted not statistically different from chance level (*p* > 0.05, Fisher’s exact test). Each stimulation ramp (slow, medium and fast) had the same time duration (T)

During the intra-digit temporal Dynamic Recognition task (DR – Fig. 2), Subject 1 was asked to recognize three different dynamics of neural stimulation injected using a single TIME channel (in the median electrode, ME). The time t characterized the intra-digit temporal dynamic for each stimulus, and it was defined as the time of the amplitude modulation necessary to reach a maximum level of sensation (S_max_ = 8/10) starting from the perceptual threshold (S_min_ = 1/10). In the ME, we modulated the amplitude of the intraneural stimulation linearly as in [10] between A_min_ = 200 μA and A_max_ = 300 μA, fixing pulse-width to 80 μs and frequency to 50 Hz [7]. We called ‘Delta_DR_’ the difference in time across the three stimuli (i.e. t_slow_ = 4 s, t_medium_ = 2.5 s and t_fast_ = 1 s, Delta_DR_ = 1.5 s) describing the slope of the amplitude increase. Each stimulation ramp (slow, medium, and fast), even with different temporal dynamics, had the exact same time duration (T) with a rising phase (rp) and a saturation phase (sp) (e.g., as in Fig. 2a slow ramp: rp = 4 s + sp. = 2 s; medium ramp: rp = 2.5 s + sp. = 3.5 s; fast ramp: rp = 1 s + sp. = 4 s). These temporal dynamics of amplitude modulation was predefined and presented randomly to the subject during DR. The subject was blindfolded and acoustically insulated, and she had to indicate vocally ‘Fast’, ‘Medium’ or ‘Slow’ dynamics. Six different values of Delta_DR_ were tested (5 s, 3 s, 2 s, 1.5 s, 0.5 s and 0.25 s). Each Delta_DR_ constituted an individual experimental session. Each session was composed of 30 repetitions per 3 different stimuli (90 repetitions in total) presented to the subjects in a random order. All the sessions were performed in the same day. Delta_DR_ was reduced across the sessions until the performance of the task had reached chance level in order to find the limit in the temporal dynamic recognition (defined as the temporal perceptual threshold).

### Inter-digit temporal distance recognition (TR)

During the inter-digit Temporal distance Recognition task (TR – Fig. 1), both Subject 1 and 2 were asked to recognize three temporal neural stimulations eliciting two separated sensations (ME and UE, Fig. 1). The time ‘t’ characterized the inter-digit temporal distance in delivering between the two stimulation trains of 10 s. We called ‘Delta_TR_’ the difference in t’ across the three stimuli (i.e. t’_ME_ = 2 s, t’_same_ = 0 s and t’_UE_ = 2 s, Delta_TR_ = 2 s). The temporal distance or stimulus onset asynchrony (SOA) [24] between the two stimuli has to exceed a specific threshold otherwise the stimuli were perceived synchronously. The SOAs were predefined and presented randomly to the subject during TR. The subject was blindfolded and acoustically insulated, and she had to indicate vocally ‘ME before’, ‘Equal’ or ‘UE before’. The subject reported ‘ME before’ when the sensation on the medial palm arrived before the one perceived on the ulnar side, ‘UE before in the opposite situation and ‘Equal’ when the inter-digit temporal distance was inappreciable. Seven different Delta_TR_ (5, 3, 2, 1, 0.5, 0.25, 0.125 s) were tested. Each Delta_TR_ constituted an experimental session. Each session was composed of 30 repetitions per 3 different stimuli (90 repetitions in total) presented to the subjects in a random order. All the sessions were performed in the same day. Delta_TR_ was reduced across the sessions until the performance of the task has reached chance level in order to find the limit in temporal distance recognition (defined as the temporal perceptual threshold).

The subjects’ performances were compared with the ones achieved by the same subject during the closed-loop experiments of Shape Recognition Task (SRT) reported by Valle et al., 2018 [10] (Fig. 4) in two different sessions. The hand tension sensors were placed in the middle and little fingers of the robotic hand. In this task, the subjects were asked to perform a single palmar grasp to explore the object (three possible shapes, as explained below), then report their perceptions and release the grasp. Since the starting position was always the same (open hand position) as well as the object position on the robotic hand, the closure velocity was similar among trials (Fig. 4 a). The three different items were: a cylindrical object (the middle and little fingers touched the object at the same time), a spherical object (the middle finger touched the object before the little finger), and a trapezoidal object (the little finger touched the object before the middle finger). First session was performed during the 2nd month and the second session during the 3rd month after implant for Subject 1. Subject 2 performed the first session in the 3rd month and the second session on the 4th month after implant. In this task, the objects were real and with a Delta_TR_ of 1.52 ± 0.3 s (Fig. 4a) calculated starting from the object’s contact (i.e. contact between a robotic digit with the object).

### Bidirectional hand prosthesis in the closed-loop experiments

During the closed-loop experiments of objects’ recognition, the subjects exploited the bidirectional hand already presented in Petrini et al., [6]. The subjects exploited a research prosthesis comprising a robotic hand with grip force sensors (Miniature tendon force sensors: Tendon Force Range = 64 N; Size = 6 mm diameter and 5 mm height; Sensor_Vcc_ = 5 V; Sensitivity|_Vcc = 5_ = 51.6 mV/N; ADC sensitivity = 0.0625 N/LSB; Hysteresis = 1.39%FSO; Linearity Error = 1.21%FSO; Repeatability = 1.56%FSO) in the middle and little fingers (IH2 Azzurra hand, Prensilia SRL, Italy), a recording and stimulation device (Neural Interface Processor, Ripple LLC, US) that acquired the muscular signals (EMG) from the surface electrodes and conveyed sensory feedback about the grip force to the subject through two channels of the TIME inserted in the median and ulnar nerves, and a single-board computer (Raspberry Pi 3, Raspberry Pi Foundation, UK) running a custom multithreaded C application that interpreted the subject’s muscle contractions to open and close the hand, and controlled the neural stimulator based on the sensors readout (Additional file 1).

In order to control the prosthesis, four surface EMG signals were acquired from the residual muscles in the forearm. Two electrodes picked up signals from the dorsal and two from the ventral side of the forearm. The signals were sampled at 2 kHz and digitally filtered using a 4th order band-pass (15–375 Hz) Butterworth IIR filter, and a notch filter to remove the 50 Hz power line interference. In particular, the prosthesis control was based on a three-state (open = − 1, close = 1, rest = 0) k-Nearest Neighbor (kNN, k = 3) classifier with a decision-based velocity ramp [25]. The classifier input was the waveform length (WL) of the EMG channel, computed over a window of 100 ms, and allowed a gated-ramp control [26] of the speed of the robotic hand movements. This means that if the patient was closing the hand in 2 different trails, by maintaining for the same amount of time the closing condition, the hand was closing in the same amount of time. The hand speed control followed this relation:

1 $$ {\mathrm{V}}_{\mathrm{hand}}\leftarrow {\mathrm{V}}_{\mathrm{hand}}+\mathrm{A}\ast {\mathrm{Class}}_{\mathrm{output}};\kern2em {\mathrm{when}\ \mathrm{Class}}_{\mathrm{output}}=-1,1; $$

2 $$ {\mathrm{V}}_{\mathrm{hand}}\leftarrow 0;\kern2.25em {\mathrm{when}\ \mathrm{Class}}_{\mathrm{output}}=0; $$

where A is a proportional factor that can be controlled by the experimenter, Class_output_ is the output of the classifier, and V_hand_ is a number in the range between 0 and 511, that controls the velocity of the motors of the robotic hand. When V_hand_ = 1 the angular velocity of the robotic hand is 0.297 deg/sec, when V_hand_ = 511 the angular velocity of the robotic hand is 511*0.297 deg/sec = 151.76 deg/sec.

During TR in closed-loop starting form an open hand position, the participants were asked to perform a continuous hand closure movement (i.e. contraction of the stump flexors muscles) until they gave the answer about the object shape. After the reply, the participants performed an hand opening movement (i.e. contraction of the stump extensors muscles) and the robotic hand was manually adjusted by the experimenter to a full open position (the new starting point).

We compared subjects’ performance in TR in experimental and simulated conditions with stimuli having similar delta (1.52 ± 0.3 s, Fig. 4a) calculated starting from the object’s contact (i.e. contact between a robotic digit with the object).

### Statistical analysis

All data were analyzed using MATLAB (R2016a, The MathWorks, Natick, US). All statistics were performed using the available built-in functions. Fisher’s exact test (p) was used to compare the performance between open and closed loop experiments. All reported *p*-values resulting from the Fisher’s exact test (p) measure the significance of the statistic. The number of repetitions for each experiment is reported in the corresponding figure captions.

## Results

Two stimulation channels (ME and UE) of TIMEs were used to deliver stimulation in the present study. Sensation location, extent, type, and intensity (S_min_ perceptual threshold and S_max_ maximal level) evoked by injecting current through ME and UE were first characterized. The sensation locations were reported by the subjects using a custom-made GUI. In particular, for Subject 1 ME elicited a ‘pressure’ sensation localized in the lateral part of the palm close to index and thumb, while UE a ‘tingling’ sensation on and below the 5th finger (Fig. 1b). For Subject 2, ME elicited a ‘vibration’ sensation on the bottom part of the palm while UE evoked a ‘vibration’ sensation on the 5th finger. As expected, a linear increase in amplitude stimulation led to an increase in the perceived intensity (Fig. 1c). In particular, in Subject 1, modulating stimulation between 200 and 300 μA with a pulse width of 80 μs and a repetition frequency of 50 Hz increased sensation intensity from 1 (out of 10, perceptual threshold) to 8/10 (maximal level). Differently, simultaneous stimulation of ME and UE (multichannel stimulation versus the common ground) led to the perception of two locally-separated sensations at the same time and intensity (S = 5/10) for both subjects. Stimulation parameters are reported in Fig. 1d.

During DR, the subject was asked to recognize three stimuli variable in intra-digit temporal dynamic (modulation between perceptual threshold and maximal level) presented randomly. The performance changed according to Delta_DR_ (difference between intra-digit temporal dynamics), particularly diminishing while Delta_DR_ was decreased (Fig. 2). The results showed that below 0.5 s between the stimuli (ST) (i.e. ST1 = 1.5 s, ST2 = 1 s and ST3 = 0.5 s), Subject 1 was not able to reach a performance above chance level (P_0.25_ s = 33.8%). The difference between performance and chance level was not statistical different (Fisher’s exact test, *p* > 0.05).

**Fig. 3:**
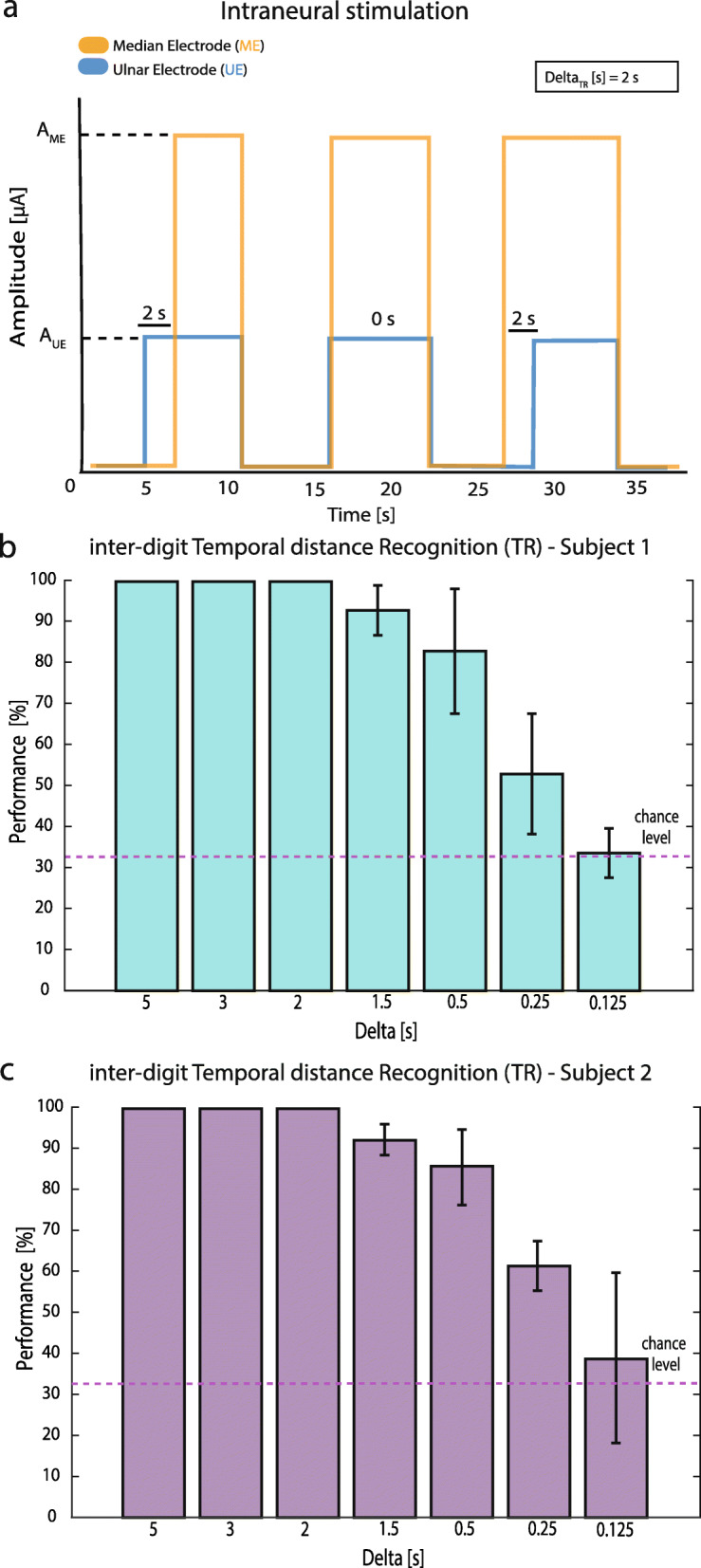
Inter-digit Temporal distance Recognition (TR) task to determine the sensitivity to inter-digit temporal distance of intraneural stimulation. **a** In TR the timing between two stimulation trains coming from two different active sites was changed. **b** Performances according to different Delta_TR_ are reported for Subject 1 (*N* = 7 sessions each of 90 repetitions). Means ± STD are reported. Delta_TR_ = 0.125 s resulted not statistically different from chance level (*p* > 0.05, Fisher’s exact test). **c** Performances according to different Delta_TR_ are reported for Subject 2 (*N* = 7 sessions each of 90 repetitions). Means ± STD are reported. Delta_TR_ = 0.125 s resulted not statistically different from chance level (*p* > 0.05, Fisher’s exact test)

During TR, subject 1 and 2 were asked to recognize three stimuli changing in inter-digit temporal distance (stimulation delivered by two channels at different times with the same intensity) presented randomly. The performance changed according to Delta_TR_ for both subjects (difference between inter-digit temporal distances), particularly diminishing while Delta_TR_ decreased (Fig. 3b,c). The results showed that below 0.25 s between the stimuli on the different channels, the subjects were not able to reach a performance above chance level (P_0.125_ s = 33.6%). The difference between performance and chance level was not statistically different (Fisher’s exact test, *p* > 0.05).

Then, we compared the performance achieved by the subjects during the TR parametric experiments in ‘open-loop’ configuration (called here “simulated” condition) with those achieved when exploited in the ‘closed-loop’ system (Shape Recognition Task - SRT [10], called here “experimental” condition, see Additional file 1). For comparing the performance, we simulated virtual objects with the similar Delta_TR_ (1.5 s). During TR, the results indicated that the simulated and experimental performance were respectively 93.6 and 83.6% for Subject 1, while 94.6 and 84.7% for Subject 2.

**Fig. 4:**
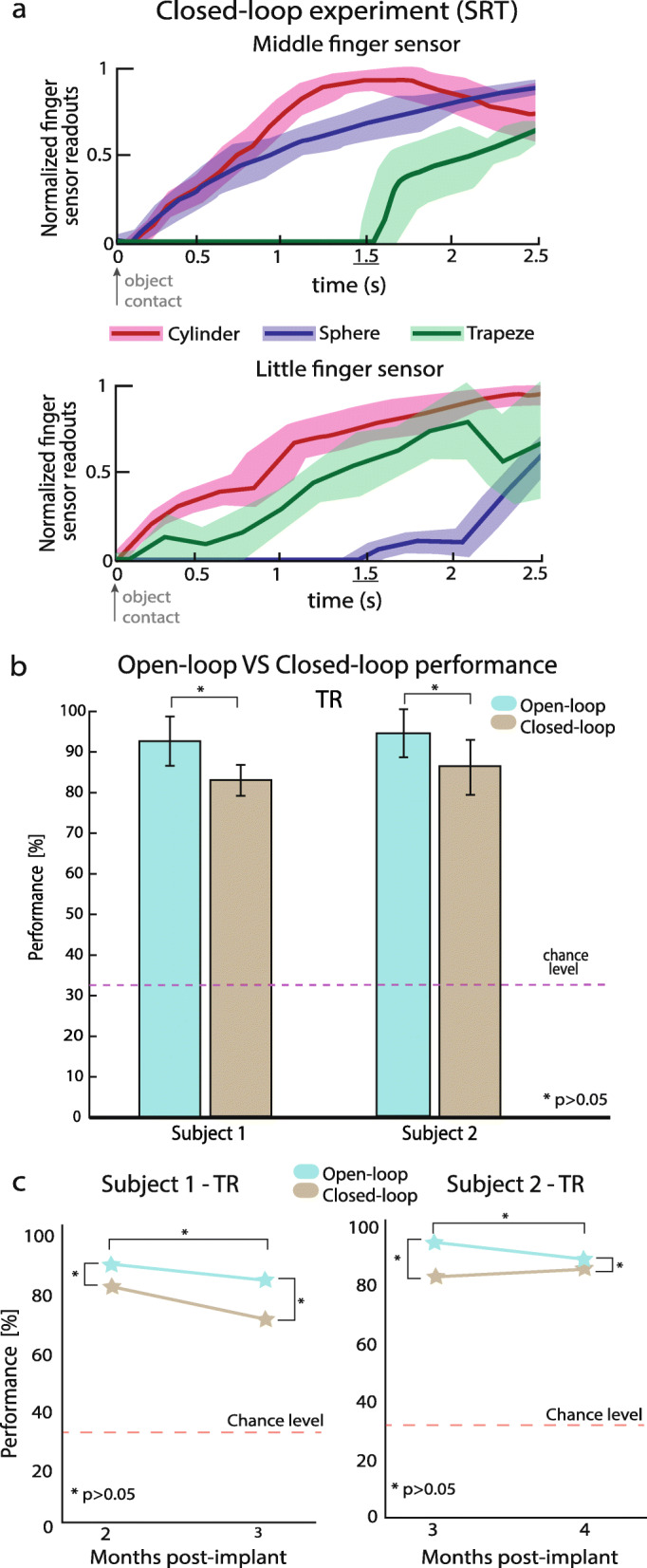
‘Open-loop’ and ‘closed-loop’ performances in TR. **a** Variability of sensor data placed on the middle and little finger of the robotic hand during the shape recognition task (SRT). The sensors’ profiles for 90 trials of Subject 1 and 2 are averages ± S.E.M. (shaded area). Profiles are plotted from threshold to saturation. **b** Comparison between open-loop and closed-loop (wearing the bidirectional hand prosthesis) performances in TR are reported for the same Delta_TR_ (1.5 s) for Subject 1 & 2. They are not statistically different (*p* > 0.05, Fisher’s exact test, 90 repetitions for each condition were considered). **c** Over time performances of open and closed loop configurations are shown for TR for both subjects. No statistical difference was found among different sessions in all conditions (*p* > 0.05, Fisher’s exact test)

They were not statistically different (Fig. 4b,c; *p* > 0.05, Fisher’s exact test).

## Discussion

This study confirmed that neural stimulation could restore, in an informative way, somatotopic sensory feedback after upper limb amputation. Indeed, the direct nerve stimulation could convey effective object characteristics thanks to the intimate contact with the sensory fibers [22] and to the correctly modulated parameters. In particular, in the current study we exploited different temporal dynamics of amplitude modulation that previously were used to convey object compliances [7, 10]. In DR, we have chosen amplitude modulation as encoding strategy, since it has been previously demonstrated to be more reliable (in terms of sensitivity and persistence) than frequency neural linear modulation [10]. In particular, using amplitude modulation it is possible to restore a higher number of perceivable intensity levels (higher sensitivity) guaranteeing a richer sensory feedback and a sensation more persistent and stable in time (higher persistence) [10].

Another characteristic that the neural modulation could encode was the object shape. Indeed, the time sequence at which the neural stimulations were delivered by different electrode sites, was related to the contact between the different contact sites of the object surface and the individual robotic digits [7, 10]. Thus, we studied the time delay necessary between two stimulation trains to identify three different temporal conditions.

Comparing the results of DR and TR, we observed that Subject 1 was able to better identify three different stimuli above chance in TR than in DR. Indeed, the time at which the performance reached the chance level was lower in TR than in DR. This evidence indicated that the intra-digit temporal dynamic is a more difficult stimulation property to understand rather than the inter-digit temporal distance in delivering stimulation through different active sites. For this reason, it is important to take into account that compliance seems to be complex to convey using direct nerve stimulation: up to now researchers have reported a maximum of three different compliances correctly discriminated by the hand amputees [4, 7, 27] even if a deeper characterization is necessary.

The consistency between the results of Subject 1 and 2 in TR task, in which the discrimination threshold is 0.125 s for both, seems to confirm the generalizability of these findings. Indeed, considering the different position of the electrode inside the nerve, it suggests that the temporal sensitivity in not necessary dependent on this process, but on spinal and/or supraspinal processes (i.e., basal ganglia [24]).

Moreover, thinking to make more realistic the identification, the subject could be asked to recognize simultaneously more than one object property combining more information encoded in the neural stimulations. Even if the perceptive thresholds of healthy subjects are lower (TR_healthy_ = 60 ms) [24, 28] than those presented here (TR_amputees_ = 125 ms) the subjects generally performed better in discriminating temporal distances than tactile temporal dynamics as the amputee exploiting neural stimulation.

Additionally, since recently it has been demonstrated that artificial intraneural stimulation was optimally integrated with visual feedback [29], the cross-modal effect of both temporal processing and multisensory integration [24] should be investigated.

Interestingly, the performance between ‘open-loop’, in which the subjects were asked to recognize different stimulation properties, and ‘closed-loop’, in which they used the prosthesis with sensory feedback grasping real objects, were not statistically different (*p* > 0.05, Fisher’s exact test). These results were stable over time, as the performance of the two subjects in TR repeated 1 month after the first session, showed no significant difference in both conditions (*p* > 0.05, Fisher’s exact test). This means that: i) the inter-digit temporal distance of the neural stimulation is well integrated in open-loop as well as in closed-loop configuration ii) the sensory recognition performance based on inter-digit temporal distance is not dependent by passive (open-loop) or active (closed-loop) configuration iii) the ‘open-loop’ stimulations are useful to determine the sensitivity and the subject’s limits in order to exploit easily the neural sensory feedback in closed-loop. Indeed, it is convenient in terms of cost and implementation to test the sensory feedback restoration without a robotic hand and real objects (with different compliances) maintaining the validity of the results. It is easier and faster to simulate at different temporal dynamics (compliance) and temporal distances (shapes) than to characterize real objects and test them (i.e. changing several object compliances maintaining same weight, texture, size and shape).

In the simulated configuration, the prosthesis control was absent, but the performances were not different from closed-loop condition. This evidence could suggest that the cognitive load due to the myoelectric control did not interfere with the sensory time discrimination, but further tests are necessary.

As shown in Raspopovic et al. [7], the patients controlling the prosthesis with sensory feedback use, to recognize shape and compliance, codes that are similar to the ones used by persons with their intact hand. For this reason (i.e., because it is a code that is previously known), the patients were able to discriminate objects without training. For the future, we will have to test whether these strategies for shape and compliance recognition can be combined to allow simultaneous recognition of more than one physical object property. It is worth noticing that in a previous work from D’Anna et al. [20], it was already shown that more than one physical object property can be discriminated simultaneously, using sensory feedback restoration through nerve stimulation by transversal intraneural electrodes.

A useful approach could be to test a large set of parameters in an ‘open-loop’ phase finding the optimal combinations. Thus, the ‘closed-loop’ configuration could be tested using a limited set of parameters, already characterized, reducing experimental time and effort.

The main limitations of the present work are: (i) our results have been achieved only with two subjects; (ii) the object shape recognition (TR) was tested in controlled conditions in which the object placement and the grasping movement were always the same not considering the variability of daily living situations; and (iii) we restrained our investigation to linear modulation of amplitude as a function of the stimulus intensity for DR. Recently, more complex feedback strategies could be envisaged, such as neuromorphic stimulations [11, 18, 30, 31] mimicking more closely the physiological behaviour of natural sensors in the skin.

In the future, it would be interesting to extend the comparative approach used in the present work to include these approaches. Since the biomimetic approach was shown to improve different aspects of artificial touch [11, 32], and to investigate whether evoking more natural sensations will improve the sensitivity to temporal information encoded in neural stimulation.

Finally, it would be of great interest to compare the results in terms of sensitivity achieved in the current study using intraneural stimulation with other stimulation modalities such as non-invasive sensory feedback approaches (e.g., TENS [33, 34]).

## Conclusions

In the present work, we evaluated the sensitivity to variations of the intraneural tactile sensory feedback temporal parameters in two trans-radial amputees, in simulated conditions (i.e. we modulated these parameters during the injection of intraneural stimulation). We found the temporal parameters thresholds for temporal dynamic and for temporal distance. Finally, we compared ‘open-loop’ performance (simulating three virtual objects) in TR with those achieved by the same subjects recognizing three different real objects in ‘closed-loop’ configuration in SRT while controlling robotic hand (experimental). The performances were not statistically different. This study is *a new* step towards a more detailed characterization of the efficacy of different intraneural stimulation parameters. This could play a pivotal rule in the optimization of this technique for an extensive clinical use in people with hand amputation.

## Supplementary information

**Additional file 1.**

## Data Availability

Data and materials used for the production of the results of the paper available from the corresponding author upon a reasonable request.

## Abbreviations

TIMEs: Transversal Intrafascicular Multichannel Electrodes
ME: Median Electrode
UE: Ulnar Electrode
SOA: Stimulus Onset Asynchrony
DR: Intra-digit temporal Dynamic Recognition
TR: Inter-digit Temporal distance Recognition
SRT: Shape Recognition Task
S_min_: Sensation at the perceptual threshold (Minimum)
S_max_: Sensation at the Maximal level
A_min_: Minimal stimulation Amplitude
A_max_: Maximal stimulation Amplitude
ST: Stimuli
